# Endoscopic Ablation in Cholangiocarcinoma

**DOI:** 10.3390/cancers17172843

**Published:** 2025-08-29

**Authors:** Cristina Natha, Varun Vemulapalli, Nirav Thosani

**Affiliations:** 1 Department of Internal Medicine, The University of Texas Health Science Center at Houston, Houston, TX 77030, USA; cristina.m.natha@uth.tmc.edu (C.N.); varun.vemulapalli@uth.tmc.edu (V.V.); 2 Department of Surgery and Interventional Gastroenterology, The University of Texas Health Science Center and McGovern School of Medicine, Houston, TX 77030, USA

**Keywords:** cholangiocarcinoma, photodynamic therapy, radiofrequency ablation, biliary stent

## Abstract

Cholangiocarcinoma is a rare, highly aggressive malignancy of the hepatobiliary tract with poor prognosis, often diagnosed at advanced stages when curative surgical resection is not feasible. Management increasingly relies on advanced endoscopic interventions to address malignant biliary obstruction and improve clinical outcomes. Beyond conventional biliary stenting, adjunctive endoscopic ablation therapies have emerged as promising strategies to improve both stent patency and survival. This review comprehensively examines the evolving role of radiofrequency ablation and photodynamic therapy in the treatment of unresectable cholangiocarcinoma. Radiofrequency ablation utilizes localized thermal energy to induce coagulative tumor necrosis and offers advantages including procedural simplicity, favorable safety profile, and cost-effectiveness; however, its efficacy may be limited by tumor size, location, and proximity to critical structures. In contrast, photodynamic therapy employs light-activated photosensitizers to selectively induce cytotoxicity in malignant tissue, demonstrating superior outcomes in prolonging both stent patency and overall survival across multiple studies and meta-analyses. Photodynamic therapy’s ability to treat more diffuse and peripheral lesions represents an important advantage, though its use is limited by photosensitivity reactions and shallow tissue penetration. Ultimately, endoscopic ablation therapies represent valuable adjunctive options in the multidisciplinary care of patients with unresectable cholangiocarcinoma. As technological advances continue and more comparative data emerge, optimized patient selection and individualized integration of these therapies hold potential to significantly improve outcomes in this challenging malignancy.

## Simple Summary

Cholangiocarcinoma is a rare, aggressive malignancy with limited curative options. Endoscopic ablation therapies such as radiofrequency ablation (RFA) and photodynamic therapy (PDT) offer promising adjunctive treatment in unresectable cases by improving stent patency and overall outcomes. This review examines the mechanisms, clinical outcomes, and limitations of RFA and PDT. Comparative data suggest PDT may offer superior survival but is limited by cost and photosensitivity. Integrating endoscopic ablation into multidisciplinary care is essential for optimizing palliative outcomes in advanced CCA. Further studies are needed to guide optimal therapy selection.

## 1. Introduction

Cholangiocarcinoma (CCA) is a rare but aggressive malignancy that originates from the epithelium of the hepatobiliary system and presents with significant challenges in diagnosis and treatment. It accounts for approximately 15% of primary liver cancers and 3% of gastrointestinal malignancies worldwide [1]. Over the past two decades, incidence rates have steadily increased, particularly for intrahepatic CCA, with regional variation influenced by environmental exposures, diagnostic capacity, and population-specific risk factors. In high-income countries, incidence remains relatively low (0.3–2 per 100,000 annually), while the highest incidence is observed in the Asian continent. Endemic regions of Southeast Asia report rates nearly 40-fold higher, driven largely by liver fluke infections [2,3]. In the United States, the age-adjusted incidence of liver and intrahepatic bile duct cancers has reached 9.4 per 100,000, with 5-year relative survival at just 22% [4].

**Figure 1:**
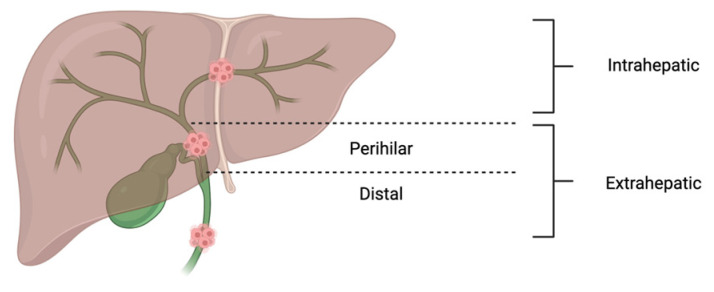
Subtypes of Cholangiocarcinoma. Perihilar CCA occurs just outside the liver, between the junction of the right and left hepatic ducts (the common hepatic duct) down to the cystic duct. Distal CCA occurs outside the liver while intrahepatic CCA occurs in the bile ducts within the liver [2,5,6,7].

CCA is anatomically classified into intrahepatic, perihilar, and distal extrahepatic subtypes, with the vast majority (~90%) of occurrences being extrahepatic (Figure 1) [2,5,6,7]. Each type has its distinct clinical behavior, resectability profiles, and molecular features. Histologically, the majority of CCAs (>90%) are adenocarcinomas, with squamous cell carcinoma being responsible for most of the remaining cases [8]. The malignancy is often characterized by dense desmoplastic stroma, perineural invasion, and vascular encasement—factors that contribute to its insidious progression and frequent late-stage diagnosis [9]. Numerous risk factors have been implicated in CCA pathogenesis. These include primary sclerosing cholangitis, cholelithiasis, genetic disorders, congenital biliary malformations (e.g., choledochal cysts, Caroli disease), viral hepatitis (B and C), alcohol-related cirrhosis, non-alcoholic fatty liver disease, diabetes, obesity, and chronic biliary inflammation [10].

The diagnosis of CCA is made through a combination of clinical, radiologic, and pathologic assessments. Patients commonly present with nonspecific symptoms such as jaundice, pruritus, abdominal pain, or weight loss. Laboratory studies often reveal a cholestatic pattern of liver injury and may include elevated tumor markers such as CA 19-9 and CEA. Cross-sectional imaging with ultrasound, computed tomography (CT), and magnetic resonance imaging (MRI) is essential for detecting masses, ductal dilation, and biliary strictures [11]. Clinical suspicion of CCA typically arises from biliary obstruction or mass lesions identified on imaging, but confirming the diagnosis requires direct tissue sampling via endoscopic retrograde cholangiopancreatography (ERCP) or endoscopic ultrasound (EUS). While brush cytology during ERCP is often the first diagnostic step due to its ease and high specificity, its sensitivity is limited. Therefore, histologic confirmation via forceps biopsy or EUS-guided fine-needle biopsy is warranted when cytologic results are equivocal or when malignancy is suspected but not confirmed. Histology is particularly important in cases where cytology is inconclusive or negative despite strong clinical and radiologic suspicion, as it allows for architectural evaluation and ancillary testing, including immunohistochemistry or molecular profiling, which may guide therapy [12,13,14].

CCA generally has a poor prognosis due to its aggressive nature and late presentation. Surgical resection remains the cornerstone of curative treatment; however, only 20–30% of patients are candidates for resection at the time of diagnosis due to locally advanced or metastatic disease [15]. Even among resected cases, recurrence is common, and 5-year survival rarely exceeds 20–50% [16,17,18]. In contrast, the five-year survival rate in unresected cases ranges between 5% and 17% depending on the location and progression of malignancy [19].

For highly selected patients with early-stage perihilar CCA and underlying sclerosing cholangitis, liver transplantation following neoadjuvant chemoradiation has demonstrated favorable outcomes and is now an accepted approach in this subset [20]. For resected cases, adjuvant chemotherapy with capecitabine remains the standard of care based on the BILCAP trial [21]. In unresectable or metastatic settings, gemcitabine plus cisplatin is commonly used for palliative treatment, with recent data supporting the addition of immune checkpoint inhibitors such as durvalumab for improved survival [22]. In parallel, the increasing use of next-generation sequencing has identified actionable mutations (e.g., FGFR2 fusions, IDH1 mutations, HER2 amplification), allowing for the use of molecularly targeted therapies in biomarker-selected subgroups [23].

Despite these systemic advances, biliary obstruction remains a common and clinically significant complication in unresectable CCA. In these cases, palliative therapies play a crucial role in improving patients’ quality of life primarily through management of biliary obstruction and the accompanying symptoms [24]. Endoscopic retrograde cholangiopancreatography (ERCP) with biliary stenting is a widely used palliative intervention for patients with unresectable cholangiocarcinoma, offering symptomatic relief through biliary decompression. However, its long-term effectiveness is often limited by stent occlusion and recurrent biliary obstruction. In response to these challenges, adjunctive endoscopic ablation therapies—particularly radiofrequency ablation (RFA) and photodynamic therapy (PDT)—have emerged as promising options in the palliative setting. These minimally invasive modalities aim to delay tumor progression, improve stent patency, relieve symptoms, and potentially enhance survival [25]. Given their favorable safety profiles and growing body of supportive evidence, RFA and PDT are increasingly recognized as valuable tools in the management of unresectable CCA.

This review will examine the role of endoscopic ablation, primarily RFA and PDT, in the palliative treatment of CCA and explore their clinical indications, comparative efficacy, and evolving integration into current therapeutic algorithms.

## 2. Endoscopic Technologies

Endoscopic technologies play a significant role in the diagnosis and management of CCA. ERCP is widely used for biliary decompression and can also facilitate tissue acquisition via brush cytology and forceps biopsy [26]. However, it is important to note that the results of ERCP-based sampling vary widely between the different modalities of testing [27,28,29,30]. For this reason, ERCP is often performed with more advanced imaging techniques such as cholangioscopy to allow for improved visualization of the biliary tract and more targeted biopsies [31]. Similarly, endoscopic ultrasound with fine-needle aspiration (EUS-FNA) offers a complementary approach, particularly useful in evaluating extrahepatic lesions and nodal involvement, although its role in staging intrahepatic disease is more limited [12,32].

While ERCP and EUS are essential for diagnosis and palliation, recent advances have expanded their utility through the integration of adjunctive ablative therapies. In particular, radiofrequency ablation (RFA) and photodynamic therapy (PDT) have emerged as promising endoscopic modalities for patients with unresectable CCA. These therapies aim to enhance the effectiveness of biliary stenting by reducing tumor burden, prolonging stent patency, and alleviating obstructive symptoms [24,26].

The following sections will examine the clinical application, comparative outcomes, and limitations of RFA and PDT in the palliative management of CCA.

### 2.1. Radiofrequency Ablation

**Figure 2:**
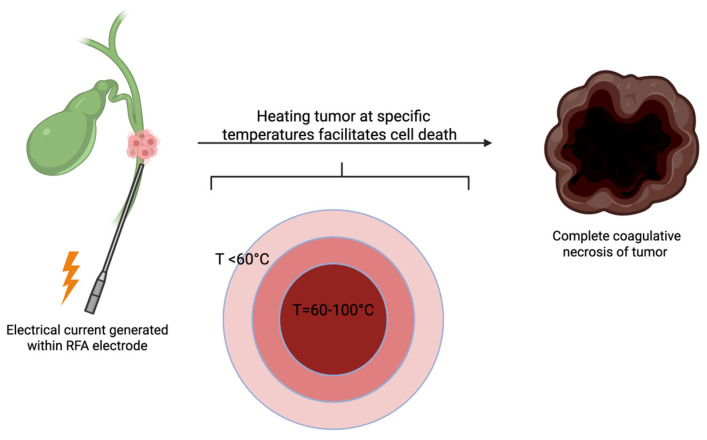
RFA Administration in Cholangiocarcinoma. RFA electrode is advanced to the site of the tumor and then heated to temperature between 60 and 100 degrees Celsius to achieve optimal cell necrosis. Cell necrosis is less optimal at tissue further from the electrode due to dissipation of heat, requiring repeated ablation at different sites to ensure complete RFA and coagulative necrosis [33,34].

Radiofrequency ablation is a minimally invasive technique that functions by delivering high frequency alternating electrical current (typically between 400 and 500 kHz) through an electrode to generate heat within the targeted tissue. This process induces heat at specific temperatures that is then transferred to tissues to facilitate coagulative necrosis of affected tumor cells. Coagulative necrosis is achieved with temperatures greater than 60 degrees Celsius, with the goal temperature being between 60 and 100 degrees depending on type of tissue, tissue conductivity, and distance of tumor cells from the electrode (Figure 2) [33]. It is important to note that incomplete RFA, whether due to tumor shape and location or due to the heat sink effect (loss of heat into adjacent blood vessels), leaves behind residual tumor which can lead to tumor progression and metastasis [34]. RFA is desirable as an endoscopic ablation technique given the benefits of the intervention as well as the low risk of adverse events. For example, Yang et al. observed no significant changes in postoperative adverse events when comparing patients who had undergone biliary stenting alone and biliary stenting with RFA [35]. Although not regarding CCA, studies on the use of RFA for hepatocellular carcinoma have further demonstrated the relative safety of this procedure with a reported procedure related mortality of 0.2% and overall complication rate of 2.2% [25]. Ultimately, the goal of RFA is to eliminate tumor cells without disrupting the surrounding healthy tissue.

#### 2.1.1. Clinical Application of RFA

The procedure for RFA involves the use of two different types of probes. The monopolar probe includes a generator, a delivery electrode, and a dispersive electrode [36]. The generator produces high frequency alternating electrical currents that travel down the delivery electrode generating heat within the probe [37]. This energy is then transferred by the delivery electrode to the dispersive electrode which disperses the energy in the target tissue to induce the coagulative necrosis or tumor cells. The second type of probe, a bipolar probe, uses two interstitial electrodes. In this configuration, the current is confined between two electrodes in close proximity without the need for a dispersive electrode [38]. The bipolar electrode has the advantage of more specific and targeted application of heating, as well as more rapid heating of the probe, which decreases the risk of injury of surrounding tissue [36]. However, this probe has also been shown to be less effective than the monopolar probe [39].

These configurations are often used in conjunction with ERCP or EUS in order to deliver the energy needed for tumor cell eradication. ERCP-guided RFA in CCA is performed by first performing wire guided biliary cannulation. Once the location and length of the stricture is determined the RFA catheter is advanced into the bile duct over the guidewire and energy is delivered to the lesion while taking caution to avoid normal tissue [40]. A stent is often placed after this intervention to maintain ductal patency. While EUS is often used in the diagnosis of CCA, EUS-guided RFA is primarily used in treatment of pancreatic malignancies rather than of CCA [41].

#### 2.1.2. Outcomes of RFA for CCA

Several studies have evaluated the efficacy of RFA and have found that there are benefits of prolongation of stent patency as well as possible survival benefits in patients with CCA receiving RFA [25]. One such study found that stent patency was prolonged in those with stents who received RFA therapy when compared to those who had stents but did not receive RFA (81% vs. 35%, *p* < 0.05) [42]. A meta-analysis conducted by Sofi et al. that included 9 studies and a total of 505 patients (239 with RFA and biliary stent and 266 with biliary stent alone) further supported these findings. This study described significant findings of prolonged stent patency of 50.6 days in the RFA group when compared to the control group. This meta-analysis also showed that there was a significant increase in median survival among the RFA group (285 days vs. 248 days) [43]. Another study on patients with unresectable distal and perihilar CCA reported similar results in which results were significant for prolonged stent patency (6.8 months vs. 3.4 months) and increased survival (13.2 months vs. 8.3 months) in those with stents who received RFA when compared to those with stents alone [35].

While there have been several other studies reporting similar results, it is important to note that there have been some studies that do not completely support these findings. For example, Wu et al. conducted a retrospective study of patients with extrahepatic CCA and biliary stents to compare those who received RFA and those who did not. The RFA group was found to have a prolonged median stent patency for uncovered self-expandable metal stents, but the median survival had no significant difference between the two groups regardless of whether the stents were covered or uncovered [44].

While these studies focus on RFA delivered endoscopically, it is important to note that RFA treatment in CCA can also be administered percutaneously. Because addressing intrahepatic and hilar malignancy is more complex than extrahepatic malignancy, percutaneous application of RFA has been used due to the potential for technical advantages and improved outcomes. Kim et al. conducted a prospective study comparing outcomes between those with percutaneous RFA with biliary stent and biliary stenting alone, but this study found no significant difference in median survival time between the two groups. However, the study did find a significant increase in stent patency in the RFA group (188 days vs. 155 days), an important factor in the management of patients with CCA [45]. Other studies have suggested that there may be a survival benefit depending on factors such as tumor size and disease stage [46].

#### 2.1.3. Complications and Limitations of RFA

While there are clear benefits to using RFA in CCA, the procedure is not without potential complications. The incidence of adverse events associated with RFA has been found to range from 5.6% to 27.1% [47]. These complications include acute cholangitis, acute pancreatitis, bleeding, and postoperative abdominal pain, among others. One study documented the most common adverse event to be post-procedure mild abdominal pain and found that post-procedure bleeding was observed to be more likely in percutaneous RFA while post-procedure pancreatitis has a stronger association with endoscopic RFA [25]. Notably, studies report endoscopic RFA to have incremental or no difference in risk of adverse events when compared to stent placement alone [48,49].

The use of RFA does also have some limitations. Despite the minimally invasive nature of RFA and the ability to precisely target malignant tissue and avoid vital structures, there is still a concern of damage to vessels that lie close to the tumor given that RFA needs to have direct contact with the tissue in order to be effective [25]. Furthermore, the efficacy RFA has been found to be inversely proportional to the size of the tumor, limiting its effects to tumors within a certain size range [50]. RFA is also contraindicated in pregnant patients, coagulopathies, or in those with cardiac devices [25].

### 2.2. Photodynamic Therapy

Photodynamic therapy is a non-thermal, localized ablative technique that works by combining a light-sensitive drug with targeted light exposure to destroy malignant cells. This procedure, similar to RFA, functions to eliminate malignant cells while sparing healthy tissue. In CCA, this alleviates symptoms of biliary obstruction and enhances quality of life. PDT is a good choice for management of malignancy given that the accumulation of light-sensitive drug in malignant tissue serves to limit the effects of PDT to this area, avoiding damage to surrounding healthy tissue. Ultimately, PDT is typically a well-tolerated therapy that has a favorable side effect profile and is a strong option for palliative therapy in CCA.

#### 2.2.1. Clinical Application of PDT

**Figure 3:**
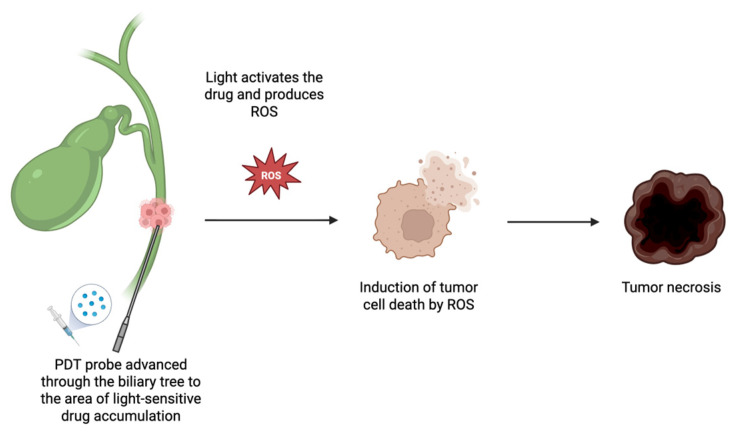
PDT Administration in Cholangiocarcinoma.

PDT is administered by first delivering an intravenous porphyrin-based photosensitizer (e.g., Photofrin^®^), which preferentially accumulates in malignant biliary tissue. Following a drug uptake period, ERCP is performed to cannulate the bile duct, and a guidewire is advanced to the lesion site. A PDT optical fiber is then introduced and positioned over the lesion. Upon activation with red light at 630 nm, the photosensitizer generates reactive oxygen species (ROS), primarily singlet oxygen, through a type II photochemical reaction [51,52]. These cytotoxic species induce localized tumor cell death via apoptosis or necrosis, depending on treatment parameters (Figure 3). Lower PDT doses and well-oxygenated tissue tend to favor apoptosis, a controlled form of cell death, whereas higher PDT doses or hypoxic tumor environments more commonly lead to necrosis, which is a less regulated and often inflammatory process [53,54,55,56].

#### 2.2.2. Outcomes of PDT for CCA

Photodynamic therapy, when combined with biliary stenting, has demonstrated significant benefits in prolonging stent patency and improving overall survival in patients with unresectable CCA. In one study, with patients with hilar CCA, the group with a biliary stent who received PDT was found to have a median stent patency of 244 days compared to 17 days in those with stenting alone. This study also demonstrated a median patient survival of 356 days in the PDT group when compared 230 days in the group with stenting alone [57]. In further support the survival benefit of PDT, one meta-analysis of ten studies showed a survival period of 413.04 days in those with biliary stent and PDT compared to 183.41 days in those with only a biliary stent [58]. Similarly, Zoepf et al. reported a median survival time of 21 months in the PDT group compared to 7 months in the biliary stent only group [59]. These studies all highlight the efficacy of PDT in prolonging stent patency for the purpose of symptomatic relief and prolonging survival, making PDT a strong option for palliative therapy in CCA.

Given that the prolongation of stent patency should reduce the symptoms of CCA, it is expected that patients experience an improvement in their quality of life after PDT treatment. Ortner et al. conducted a study which, in addition to finding that overall survival improved after PDT, found that physical functioning (measured by the Karnofsky index) and quality of life metrics (measured by QLQ-C30) also significantly improved [60]. Since PDT in CCA is a palliative treatment, this assessment is important to make to ensure that the primary objective of the therapy is being met.

#### 2.2.3. Complication and Limitations of PDT

The most significant side effect associated with PDT is phototoxicity. In the meta-analysis of ten studies previously referenced, 10.51% of those who received PDT had photosensitivity reactions, although these were self-limiting [58]. Other studies have estimated an incidence for photosensitivity of up to 25% [38]. Additionally, patients are advised to avoid sunlight for 4–6 weeks after the procedure to avoid skin reactions which can be a significant hindrance on an individual’s daily activities [37]. While cholangitis is another known complication of this intervention, several studies have observed no increased risk of cholangitis when comparing PDT/stent groups and stent only groups.

One of the most notable limitations of PDT is its shallow penetration. Because typically used photosensitizers are limited by the penetration depth of activating light, PDT has been found to be less effective when targeting deep tumors. For this reason, the use of higher energy sources has been a topic of study to overcome this issue [61,62].

### 2.3. Comparison Between RFA and PDT

While studies have shown both RFA and PDT to both be effective options for palliative therapy in CCA, there is a lack of head-to-head trials comparing the treatments. This is likely because given the low incidence of CCA, most studies comparing PDT and RFA consist of small cohorts while studies consisting of larger cohorts are typically reviews and meta-analyses. Larger reviews and meta-analyses do suggest that PDT may be more effective in prolonging survival and stent patency in individuals with CCA. For example, Mohan et al. conducted a review and meta-analysis of 55 studies involving 2146 patients (1149 who received PDT, 545 who received RFA, and 452 who received stenting alone). This study found that the overall survival rate was 11.9 months with PDT, 8.1 months with RFA, and 6.7 months with biliary stenting alone. PDT also had a mortality rate of 3.3% compared to 7% in patients who received RFA [63,64]. In contrast to this study, Strand et al. conducted a study of 48 patients (16 who received RFA and 32 who received PDT) and reported a median survival of 9.6 months in the RFA group and 7.5 months in the PDT group [65]. However, this finding was not statistically significant (*p* = 0.799). Another study, a meta-analysis including 2 studies and a total of 105 patients (49 received RFA and 56 received PDT), reported a median survival of 11.3 months in the RFA group and 8.5 months in the PDT group (*p* = 0.02) [66].

Ultimately, these studies demonstrate that while PDT may be superior to RFA in overall survival and stent patency, enough clear evidence does not yet exist to definitively make this conclusion. However, it is likely that RFA and PDT are both superior to stenting alone in overall survival and stent patency (Table 1).

Given that PDT and RFA have both been shown to increase overall survival and stent patency when compared to biliary stenting alone, it is important to recognize when to use each of these therapies. The most important factor to consider when deciding which therapy to use is the safety profile, specifically the risk of photosensitization with PDT. Given the incidence of photosensitization after PDT use, this therapy should be used with caution. Nanashima et al. outlined their proposed nursing care protocol to be followed after PDT administration for digestive tract cancers [67]. This protocol consisted of dermatology consultation, maintaining darkness in patient rooms, skin protection when leaving dark areas, and other interventions for skin protection. Even after discharge, patients required staying in dark rooms, wearing protective clothing and sunscreen when going outside, and weekly outpatient follows ups for 1 month after discharge to ensure no adverse effects. This protocol requires meticulous care and significant resources that are not required for patients post-RFA. The lack of photosensitization in RFA is the most significant and clinically relevant advantage it has when compared to PDT (Table 1).

Therapy selection for patients should also consider tumor characteristics and cost-effectiveness. The key tumor characteristic when deciding on the optimal therapy is tumor location and access. Because PDT delivers treatment by a guidewire that can be advanced through the whole biliary system, it is more easily able to treat peripheral and difficult to reach lesions. RFA, on the other hand, requires the lesion to be reachable in order to be ablated, limiting its effectiveness in intrahepatic CCA [25,63]. In regard to the cost of RFA and PDT, RFA has a significant advantage. An RFA catheter costs approximately USD $1295 while the cost of a single dose of porfimer sodium (Photofrin^®^), the photosensitizing drug used in PDT, is USD $37,208 [68]. In clinical practice, this drastic difference in cost not only affects a patient’s ability to afford a specific treatment but also affects the availability of these treatments at medical centers as RFA is typically more available and more commonly employed [69]. Further studies should be performed to determine the cost-benefits of PDT and RFA (Table 1).

## 3. Other Ablation Therapies for CCA

While RFA and PDT are the most common endoscopic ablation techniques for management of CCA, other lesser used modalities have also shown promise. One such therapy is intraluminal brachytherapy (ILBT), an internal radiation therapy technique used as a palliative treatment for patients with unresectable CCA, particularly those with malignant biliary obstruction. This procedure involves the placement of a radioactive source directly within the bile duct via a catheter, often following endoscopic or percutaneous stenting. ILBT delivers high-dose radiation locally to the tumor while minimizing exposure to surrounding healthy tissues, thereby offering a targeted approach to tumor control. It is typically used in combination with external beam radiation therapy (EBRT) or chemotherapy to enhance treatment efficacy. ILBT has been shown to improve local tumor control, prolong stent patency, and relieve symptoms such as jaundice and pruritus, thereby enhancing patients’ quality of life. Several studies have suggested that patients receiving ILBT in addition to biliary drainage may experience longer median survival compared to drainage alone. While ILBT is a promising new therapy, it requires careful planning and execution due to the risk of radiation-induced complications, such as cholangitis, strictures, or ulceration of adjacent structures. Further research is ongoing to optimize dose schedules and assess long-term outcomes of this promising modality [70].

Another therapy for management of CCA is percutaneous microwave ablation therapy (MWA). MWA is a newer thermal ablation technique used for the treatment of solid tumors such as hepatocellular carcinoma, liver metastases from colorectal carcinoma, and less commonly for pancreatic tumors. MWA is similar to RFA in that it also works by generating heat and inducing coagulative necrosis; however, it does so by using electromagnetic energy rather than an electrical current [71]. Compared to RFA, MWA allows for more rapid and homogenous tissue heating, higher intratumoral temperatures, and larger ablation zones. Percutaneous MWA is important because, similar to percutaneous RFA, it has more potential for treating intrahepatic CCA than endoscopically administered RFA and PDT [71]. While current data on MWA in intrahepatic CCA is limited, current studies have shown it to be a safe and effective alternative therapy in managing this malignancy when resection is not feasible [71,72]. Furthermore, a study comparing those who had MWA and liver resection for treatment of intrahepatic CCA found MWA to have similar overall 5-year survival (44.8% vs. 40.4%), positioning MWA as a potential alternative to liver resection when adequate margins for MWA are obtainable [73].

## 4. Innovation in CCA Treatment

Immunotherapy, specifically immune checkpoint inhibitors (ICI), has recently been shown to be effective in CCA. While ICI monotherapy has proven to be useful in malignancies such as lung cancer, kidney cancer, and liver cancer, its effectiveness in CCA is limited [74]. However, ICIs used in combination with chemotherapy have yielded better results.

The TOPAZ-1 trial demonstrated that a regimen of durvalumab with gemcitabine and cisplatin in those with advanced biliary tract cancer showed an overall survival benefit. In the updated results of this trial, the group treated with durvalumab plus gemcitabine and cisplatin had a median overall survival of 12.9 months (95% CI 11.6–14.1) compared to 11.3 months (95% CI 10.1–12.5) with chemotherapy alone. The group treated with this regimen was also found to have a Kaplan–Meier-estimated-24-month overall survival of 23.6% (95% CI 18.7–28.9) when compared to the chemotherapy group 11.5% (95% CI 7.6–16.2) [75]. Furthermore, the KEYNOTE-966 trial showed a similar survival benefit when using pembrolizumab along with gemcitabine and cisplatin. The results of these trials led to the FDA approval of both regimens for the treatment of unresectable or metastatic biliary tract cancers [76].

Another potential option for CCA treatment is the combination of ablation techniques and ICI therapy. While direct evidence in CCA is limited, this approach is supported by data from other solid tumors, such as pancreatic cancer. RFA induces local tumor necrosis, releasing tumor-associated antigens and altering the tumor microenvironment (TME). These changes may enhance immunogenicity and sensitize tumors to ICIs [77].

One key mechanism involves upregulation of PD-L1 expression on neoplastic cells and tumor-infiltrating immune cells following RFA [78]. PD-L1 inhibitors, such as durvalumab and pembrolizumab, target this axis by blocking PD-L1–PD-1 interaction, thereby reversing T-cell exhaustion and promoting anti-tumor immunity. In pancreatic cancer, this has been shown to augment the local and systemic immune response, including potential activation of the abscopal effect, in which local tumor destruction releases tumor-associated antigens that stimulate a systemic immune response and lead to regression of metastatic cancer [78]. When applied in pancreatic cancer after ablation, this systemic immune response has been observed to treat metastatic cancer at sites distant from the initial region of ablation and even potentially increase overall survival, a theory also supported in mice models [41,79]. Although such synergy has not been confirmed in CCA, the immunomodulatory effects of RFA may similarly enhance responsiveness to ICIs, warranting further investigation into their combined use. Additionally, given the prevalence of PDT use in CCA already, future studies should also investigate the efficacy of PDT used in combination with ICIs.

## 5. Conclusions/Future Directions

In conclusion, CCA is a malignancy characterized by its aggressive nature, late presentation, and limited curative therapies. With the majority of cases being characterized as unresectable disease and having a poor prognosis, palliative therapies are a critical component of patient care. Endoscopic technologies, particularly ERCP and EUS, have had a significant effect on the diagnostic and therapeutic landscape of CCA, allowing enhanced tissue visualization and sampling as well as introducing palliative interventions targeted at symptomatic relief and survival benefits in cases of unresectable malignancy.

Among these palliative interventions, RFA and PDT have established themselves as significantly beneficial adjunctive treatments to biliary stenting. Both modalities function by inducing tumor necrosis—RFA via thermal energy and PDT via photochemical reactions—while attempting to preserve the surrounding tissue. Studies have repeatedly shown that these interventions are effective in prolonging stent patency and overall survival in individuals with unresectable disease.

Although comparative analyses suggest that PDT may be more effective than RFA in prolonging stent patency as well as overall survival, the distinct advantages and limitations of each therapy demonstrates their roles in clinical practice. Ultimately, integrating the use of endoscopic ablation therapies into care plans for patients with unresectable cholangiocarcinoma is vital part of management of this condition and further studies should be performed to determine the effectiveness and role of other ablative techniques.

## Figures and Tables

**Table 1:** Comparing Photodynamic Therapy and Radiofrequency Ablation.

Feature	Photodynamic Therapy (PDT)	Radiofrequency Ablation (RFA)
Mechanism	Light-activated photosensitizer generates reactive oxygen species and induces tumor necrosis	High-frequency electrical current produces localized thermal coagulative necrosis
Invasiveness	Minimally invasive (endoscopic delivery via ERCP)	Minimally invasive (endoscopic via ERCP or percutaneous)
Tumor targeting	Selective photosensitizer uptake in tumor tissue, spares healthy tissues	Requires direct probe contact
Efficacy (stent patency)	Often superior in prolonging stent patency	Prolongs stent patency but generally less than PDT
Efficacy (survival benefit)	Often with greater survival benefit	Survival benefit observed but generally less than PDT
Complications	Photosensitivity reactions (must avoid sunlight for 4–6 weeks), cholangitis	Post-procedure pain, cholangitis, pancreatitis or bleeding
Limitations	Limited depth of penetration, photosensitivity precautions needed	Risk of thermal injury to adjacent structures, less effective for larger tumors
Availability	Requires special drugs and light source, limited to experienced centers	More widely available and easer to implement
Cost	High (porfimer sodium ~$37,000 per dose + equipment)	Lower cost (catheter ~$1295); more cost-effective in most settings
